# Gas Chromatography – Ion Mobility Spectrometry as a tool for quick detection of hazardous volatile organic compounds in indoor and ambient air: A university campus case study

**DOI:** 10.1177/14690667221130170

**Published:** 2022-10-05

**Authors:** Pedro Catalão Moura, Valentina Vassilenko

**Affiliations:** 1 Laboratory for Instrumentation, Biomedical Engineering and Radiation Physics (LibPhys-UNL), 119482 NOVA School of Science and Technology, NOVA University of Lisbon, Caparica, Portugal; 2 NMT, S. A., Caparica, Portugal

**Keywords:** Air quality, ambient air, Gas Chromatography, indoor air, Ion Mobility Spectrometry, volatile organic compounds

## Abstract

Society’s concerns about the citizens**’** exposure to possibly dangerous environments have recently risen; nevertheless, the assessment of indoor air quality still represents a major contemporary challenge. The volatile organic compounds (VOCs) are among the main factors responsible for deteriorating air quality conditions. These analytes are very common in daily-use environments and they can be extremely hazardous to human health, even at trace concentrations levels. For these reasons, their quick detection, identification, and quantification are crucial tasks, especially for indoor and heavily-populated scenarios, where the exposure time is usually quite long. In this work, a Gas Chromatography – Ion Mobility Spectrometry (GC-IMS) device was used for continuous monitoring indoor and ambient air environments at a large-scale, due to its outstanding levels of sensibility, selectivity, analytical flexibility, and almost real-time monitoring capability. A total of 496 spectra were collected from 15 locations of a university campus and posteriorly analysed. Overall, 23 compounds were identified among the 31 detected. Some of them, like Ethanol and 2-Propanol, were reported as being very hazardous to the human organism, especially in indoor environments. The achieved results confirmed the suitability of GC-IMS technology for air quality assessment and monitoring of VOCs and, more importantly, proved how dangerous indoor environments can be in scenarios of continuous exposure.

## Introduction

The evaluation of volatile organic compounds (VOCs) toxicity and the assessment of air quality in both indoor and outdoor environments are very important and trending topics nowadays. Rapid and accurate detection, identification, and quantification of the VOCs are mandatory issues for the mitigation of the possible consequences that continuous exposure may cause to human health.

VOCs are regular organic compounds that present a special idiosyncrasy. Their main molecular structure is composed of carbon atoms covalently linked to hydrogen, oxygen, or other elements, as for any other organic compound, but they have the specificity of being volatile at room temperature. For these reasons, VOCs can easily traverse biological membranes like pulmonary/alveolar, ocular, and cutaneous tissues. In addition, they range from practically inert to very reactive, a fact that classifies them as potentially toxic and especially hazardous to human beings even at trace concentration levels.^1,2^

Some of the most well-known examples of VOCs are ethanol, ammonia, and acetone, due to their widespread use in daily activities.^3^ Others, like toluene and xylenes, are equally known but deeper studies regarding the possible consequences of their presence in the air on human health are still required. Despite being more or less known, VOCs are important air pollutants for both indoor and outdoor environments and are directly responsible for the development of numerous pathologies. For instance, the presence and accumulation of VOCs contribute to the syndrome of “sick building”.^4,5^ This syndrome is the main factor for the manifestation of allergic, inflammatory, and respiratory diseases. Illnesses like eye allergy, skin irritation and respiratory problems (asthma, chronic obstructive, pulmonary disease (COPD), and several others) are the most frequent health conditions in people continuously exposed to the indoor air of “sick buildings”.^4,6,7^ In more complex scenarios, VOCs like benzene and formaldehyde, have even been identified as carcinogenic.^1,8–13^

The sources and origins of VOCs are multiple and diverse. They can be released into the air by quotidian elements, such as furniture or clothes, by building materials, like paintings or coatings, by cleaning products, namely, detergents or pesticides, by personal care products, like creams or perfumes, or even by daily activities, like cooking or smoking.^9,14–16^ This fact proves how effortlessly the indoor and outdoor air can be replete with hazardous compounds so, the monitoring of their presence is crucial. Considering a hospital environment, for example, the air quality assessment is critical since the presence of VOCs can have direct consequences on the health of both medical staff and patients under treatment. An eventual continuous exposure to the VOCs existing in the air may, not only, worsen the diseases of the patients, but also, lead to the development of additional health conditions.^17,18^ In another context, public and heavily populated spaces should also receive further attention to what the air quality concerns. The presence of VOCs, at toxic levels of concentration, in kindergartens and primary schools may represent an unnecessary danger for younger children. Taking into consideration the fact that the pulmonary and respiratory system of children is under development, possible health problems at such young ages can be even more critical.^1,19–22^ The same considerations can be applied to public spaces like train and bus stations, airports, malls, restaurants, stores, and other heavily-populated locations. These locations require a correct and accurate air quality assessment in order to prevent social health problems and minimize the risks of large-scale diseases.^23–25^ Work locations where the employees**’** exposure to VOCs occurs on a daily basis, such as factories, production lines, painting lines and large-scale facilities, are also scenarios of interest and should have protocols for air quality monitoring and control in order to mitigate potential health risks during the work shifts.^26–31^

The indoor and outdoor air quality assessment involves several distinct scientific fields, such as chemistry, medicine, biology, physics, civil engineering and even architecture. Due to its complexity, reliable, sensitive, precise, and accurate devices are required for the identification, quantification, and toxicity evaluation of the VOCs. Chromatographic techniques like Gas Chromatography (GC) and High-Performance Liquid Chromatography (HPLC) are examples of largely applied technologies of detection and separation. Spectroscopic and spectrometric technologies have also been largely used, namely, Infrared Spectroscopy (IRS) and Mass Spectrometry (MS). Despite their characteristics, both techniques present some limitations regarding the assessment of VOCs. IRS often requires additional procedures for sample preparation before the analysis and its use is limited in scenarios of *in-situ* air assessment. The MS, in its turn, is not always suitable for the identification of organic molecules since its usual 70 eV radiofrequency ionisation potential frequently breaks the atomic bonds in organic compounds, inhibiting their detection. In addition, the lack of portability is, equally, a negative feature of MS.^32–34^

Regarding the aforementioned facts, a Gas Chromatography – Ion Mobility Spectrometry (GC-IMS) device was the analytical technique selected to accomplish the main goal of this work, to assess the air quality of several indoor locations from a heavily-populated university campus. Most of these correspond to locations where the local workers spend large periods of their days and the majority of the activities conducted in these spaces are potential sources of hazardous VOCs so, the air quality assessment is a mandatory and contemporary issue.

### Gas chromatography - Ion Mobility Spectrometry

Initially developed to detect chemical warfare agents and illicit drugs, in the military context, it was soon realized that IMS would also fit a variety of distinct civil applications.^23,35^ Some of those applications include health assessment,^36^ security purposes,^35^ food quality and spoilage,^37^ and even product identification and fraud assessment.^38^ If coupled with a chromatographic column, the resulting device merges the good precision, wide dynamic concentration range and high selectivity of the GC, with the outstanding sensitivity, instrumental simplicity, analytical flexibility, portability and almost real-time monitoring capability of the IMS. The improved capacities of the GC-IMS enable an accurate differentiation of VOCs based on their size, weight and molecular shape.^2,39,40^

A complete GC-IMS measurement is initiated with the injection of a gaseous sample into de device. The sample suffers pre-separation by GC where the compounds are separated by their capacity to adsorb and desorb to the inner surface of the chromatographic column. The time required for the analytes to elude from the chromatographic column is called retention time (r_t_) and it corresponds to one of the three coordinates represented in the final spectrum.^39–41^

Once pre-separated, the analytes are transferred to the IMS section of the apparatus.^42^ The working principle of an ion mobility spectrometer is based on the scientific evidence that an organic molecule, when ionised by proton transfer reaction, can be accelerated if exposed to an electric field, at ambient pressure.

Tritium (^3^H) was the ionisation source in this study, however, nickel-63 (^63^Ni) and X-ray are also commonly employed sources.^43^ The tritium source spontaneously emits β**^−^** particles that react with the inert gas inside the IMS ionisation chamber (usually nitrogen (N_2_) or simply purified air). The reaction creates background ions from the nitrogen atoms, also known as primary ions.^44–46^

Once formed, the primary ions take part in a series of new reactions with molecules of H_2_O, NH_3_ or NO present in the interior of the IMS tube and, as a result, new ions, known as reactant ions, are formed. The reactant ions are (H_2_O)_n_H^+^, (H_2_O)_n_NH_4_^+^ or (H_2_O)_n_NO^+^, respectively. Primary nitrogen ions are not represented in the final spectrum due to their very short life, but the reactant ions do appear in the spectrum. They form an intense peak visible along the entire spectrum named as reactant ion peak (RIP).^47,48^

The reactant ions are directly responsible for ionising the analytes of the injected sample. Assuming, as an example, that the sample is constituted by a volatile organic compound arbitrarily denominated M, the ionisation by the reactant ions follows equation 1.^40,49^

(1) $$M+(H_{2}O{)}_{n}H^{+}↔M(H_{2}O{)}_{n-x}H^{+}+xH_{2}O$$

During the reaction, new ions (product ions) are formed. Water molecules are formed simultaneously (x value depends on humidity levels). The product ions are protonated monomers of M since they correspond to a chemical connection between a proton and a molecule. In a scenario in which the concentration of M is more elevated, the monomer can undergo a second reaction with the remaining M molecules (equation 2).^40,49^

(2) $$M+M(H_{2}O{)}_{n-x}H^{+}↔M_{2}(H_{2}O{)}_{n-(x+i)}H^{+}+iH_{2}O$$

In these cases, the product ions correspond to protonated dimers of M. New reactions leading to the formation of protonated trimers and even larger clusters are rare but possible.^44,49–51^

The complete process described in the preceding paragraphs is known as the IMS positive mode of working, however, the spectrometer can be operated in the negative mode (normally used for more specific purposes like the detection of explosives and chemical warfare agents).^50^

For the negative mode, the formation of reactant ions occurs due to reactions of primary ions with O_2_ molecules instead of H_2_O, NH_3,_ and NO molecules, like in the positive mode. In this case, the negative reactant ions have the form of (H_2_O)_n_O_2_**^−^** or (H_2_O)_n_(CO_2_)_m_O_2_**^−^**. Considering an arbitrary volatile organic compound denominated MX, the negative product ions are formed through dissociative and associative electron attachments, as respectively represented in equations 3 and 4.^40,44,47,50^

(3) $$MX+(H_{2}O{)}_{n}O_{2}^{-}↔M+X^{-}+nH_{2}O+O_{2}$$

(4) $$MX+(H_{2}O{)}_{n}O_{2}^{-}↔(MX)O_{2}^{-}+nH_{2}O$$

Once formed, the product ions are exposed to a weak homogeneous electric field. The field accelerates the ions through the IMS tube until they eventually collide with a neutral molecule of the inert gas present in the tube. After that collision and if still under the influence of the same electric field, the ions accelerate again until a new collision occurs. This sequence of accelerations and collisions between the ion and the neutral molecules leads the product ion to tend to a constant velocity, denominated by drift velocity (v_d_).^40^ The relation between this velocity and the magnitude of the electric field (E) is called the ion mobility constant (K) of the analyte (equation 5). The drift velocity is an ion-specific value and can also be represented by the ratio between the drift length (L) and the time it takes for the ions to go through that length, denominated drift time (t_d_). The drift time corresponds to the second coordinate of the final spectrum and, like the drift velocity, it is an ion-specific value.^49^

(5) $$K=\frac{v_{d}}{E}=\frac{L}{E.t_{d}}$$

Since the ion mobility constant depends on pressure and temperature conditions, it is common to normalize K to standard environmental values of pressure (P_0_ = 760 Torr) and temperature (T_0_ = 273.15 Kelvin), obtaining the normalized ion mobility constant, K_0_, (equation 6).^35,52–54^

(6) $$K_{0}=K\frac{P}{P_{0}}\frac{T_{0}}{T}$$

**Figure 1.**
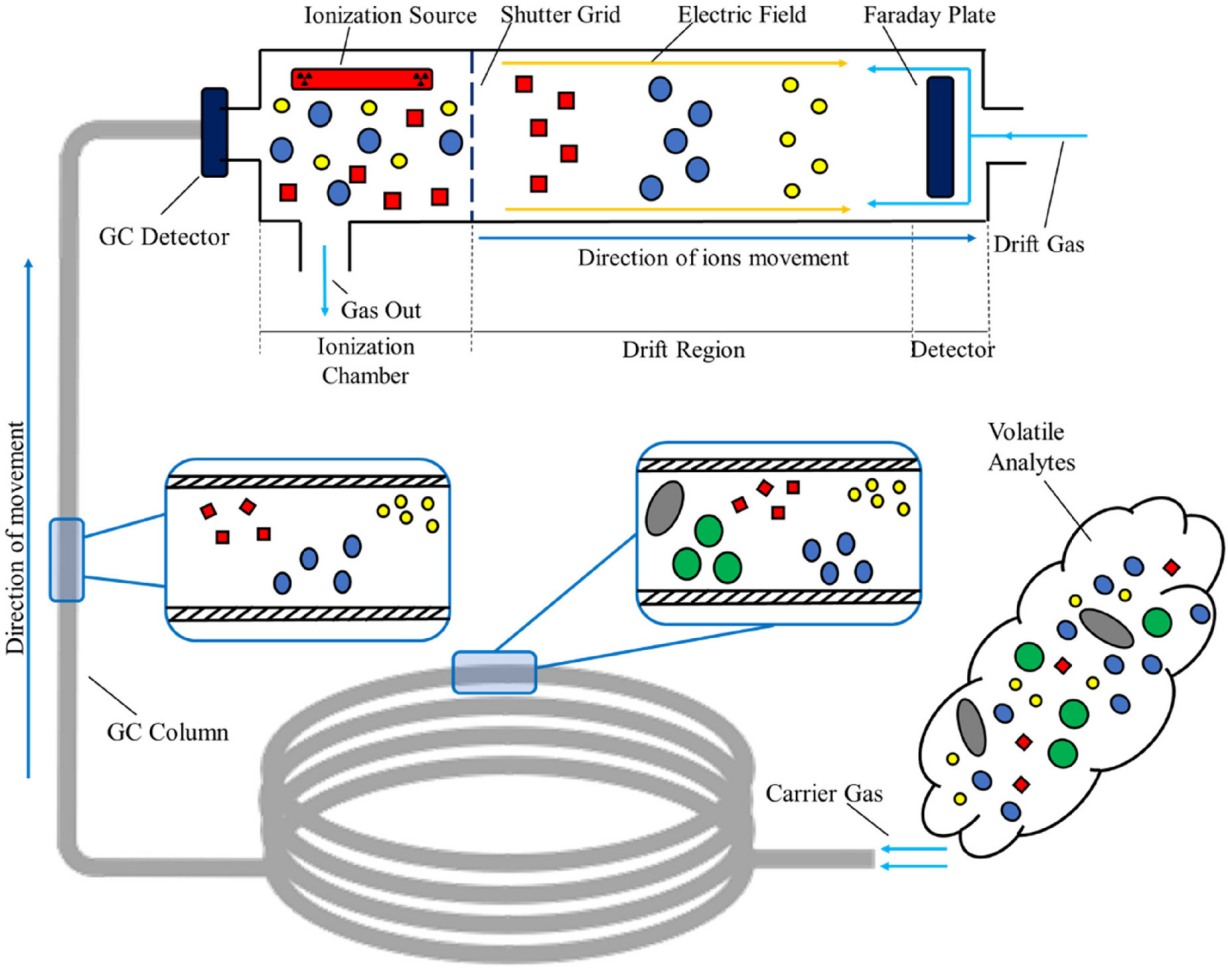
Schematic of a complete Gas Chromatography – Ion Mobility Spectrometry measurement.

Figure 1 schematizes a complete GC-IMS analysis. Yellow, blue, and green circles are the constituent compounds of an arbitrary sample injected into the GC tube. Here, they undergo a pre-separation process and then, they enter the ionisation chamber where they are ionised by the tritium source. Finally, the product ions are exposed to an electric field and, in the end, they reach the detector (Faraday plate) at different and specific drift times.

**Figure 2.**
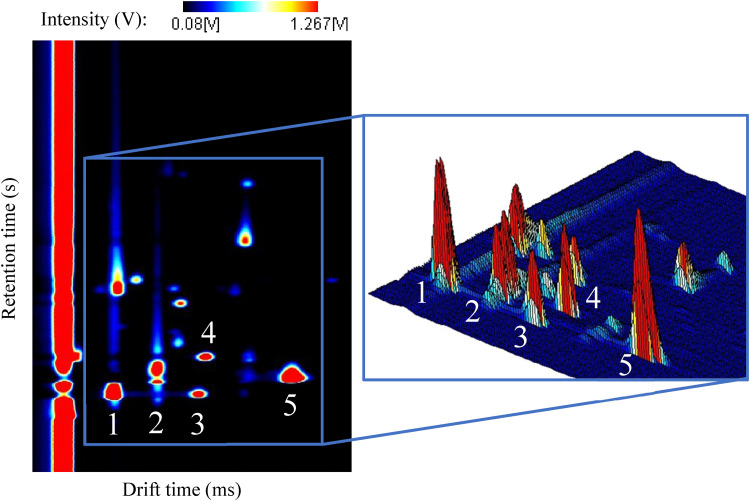
Typical GC-IMS spectrum with an enlarged section for better visualization of the three-dimensional peaks. The peaks marked with the numbers 1 and 3 correspond to the monomer and dimer of ethanol. The monomer and dimer of 2-propanol are respectively marked with numbers 2 and 5. The number 4 marks the acetone peak.

A GC-IMS measurement produces a three-dimensional spectrum as an outcome. Usually, the x-axis represents the drift time (d_t_), in milliseconds (ms), and the y-axis represents the retention time (r_t_) in seconds (s). The third coordinate corresponds to the intensity and it is represented in volts (V) along the z-axis of the spectrum. The intensity translates the concentration of each analyte in the initial sample. Figure 2 illustrates a typical GC-IMS spectrum in both two- and three-dimensional views. For the two-dimensional view, a colour scheme was used to illustrate the third coordinate, i.e., the intensity values. Ion mobility spectra can be visually interpreted taking into consideration several details of the aforementioned IMS working principle. The long red column across the entire spectrum corresponds to the RIP. Every single peak corresponds to a specific and independent volatile organic compound. Each analyte has its own specific values of drift and retention times. The retention time corresponds, as mentioned, to the time it takes for the compound to elude from the chromatographic column, so it is defined before the existence of monomers and dimers. The formation of monomers and dimers, as seen, occurs only in the ion mobility drift tube and, being subsequently produced, the monomer reaches the Faraday detector before the dimer so, they have the same retention time but different drift time. For elucidation purposes, five peaks were marked in the spectrum; the monomer and dimer of ethanol were respectively marked with the numbers 1 and 3, the numbers 2 and 5 correspond to the monomer and dimer of 2-propanol, and acetone is marked with the number 4.

## Materials and methods

### Air samples information

The study here described was conducted at a university campus^1^ located in an ecologically clean area, near the coast of the Atlantic Ocean and far away from industrial areas, meaning that the outdoor air was significantly free from toxic and exogenous contaminations. In addition, the ambient air around the campus (30 hectares) is very homogeneous and, normally, its endogenous VOCs are considerably more diluted than they are at any indoor location, they have lower concentration levels so, they can be utilized as a baseline or reference spectrum for assessing the VOCs in the studied locations. In this way, 31 air samples were collected in the outdoor environment of the campus and used as a baseline, as detailed in due course.

In order to assess the profile of the VOCs existing in the indoor air environments, 465 samples were collected from 15 different locations across the university campus. The locations were: Administration Building, Mechanical Engineering Building, Electronic Engineering Building, Laboratory of Atomic and Molecular Physics, Laboratory of Biomedical Engineering, Chemistry Laboratory, Laboratory of Analytical Instrumentation (GC-IMS), Laboratory of Electronics, Laboratory of Materials Engineering, Laboratory of Conservation and Restoration department, FABLAB – Fabrication Laboratory & 3D Printing, Canteen, Bathroom, Workshop for scientific equipment, and Storeroom for cleaning products.

The sampling sites were selected having into consideration several topics, namely, their characteristics, specific activities developed at the location, particular smells, the use of specific chemical compounds, and people's affluence, among others. For instance, the analysed buildings, like the administration, the mechanical engineering and the electronic engineering buildings, were considered for the study due to the elevated affluence of people. Heavily-populated locations must always be assessed regarding the air quality, as already mentioned. Regarding the studied laboratories, the activities developed at this type of location, usually involving chemical compounds and many other potential sources of VOCs, and the long periods of exposure that the employees have to deal with were the decisive factors to select them for analysis. The canteen was selected due to the affluence of people and the activities developed, namely, cooking. The presence of a large number of chemical products and solutions was the reason to equally select the storeroom.

### Air collection method

All the air samples were collected into a 1-litre volume chemically inert Teflon cylinder equipped with a pump and an accoupled stopcock valve. The samples were always collected within the same temperature range (19–21**°**C) and the transfer time of the cylinders from the collection site to the analytical equipment was as short as possible (in any case, not superior to 5 min). This method was drawn aiming to preserve the original quality of air samples, avoid any alterations in the temperature and humidity of the sample, and prevent any kind of interaction with exogenous compounds. Air samples were collected and analysed without any chemical or pre-concentration preparation since the GC-IMS equipment enables direct analyses and does not require any pre-processing of the sample.

### GC-IMS analysis

The GC-IMS device used during this study, named BreathSpec®, was produced by G. A. S. Dortmund. The apparatus was equipped with a 300 MBq ionisation source of tritium (H^3^ – β radiation). A 98 mm length drift tube with a 5** **kV switchable polarity was equally used. The intensity of the employed electric field intensity was 500 V/cm. The chromatographic column assembled in the device was an MXT-200 model with 30 m of length and 0.53 mm of internal diameter coated with stainless steel with a mid-polar stationary phase of trifluoropropylmethyl polysiloxane with a thickness of 1 μm. The complete list of parameters and features of the device utilised for the analyses are summarized in Table 1.

**Table 1.** Parameters and features of the GC-IMS device.

Parameters	Values	Unis
Sample Loop Volume	1	mL
GC Column Model	MXT-200	--
GC Column Length	30	M
GC Column Diameter	0.53	Mm
GC Temperature	343.15	K
Gas Nature	Purified Air	--
Carrier Gas Flow	10	mL/min
Drift Gas Flow	150	mL/min
Ionisation Source	Tritium – β Radiation	--
Ionisation Intensity	300	MBq
Ionisation Polarity	Positive	--
Drift Region Length	9.8	Cm
Drift Potential Difference	5	kV
IMS Temperature Range	297.15–301.15	K
IMS Pressure Range	757–760	Torr
Electrical Field Intensity	500	V/cm
Resolving Power Range	65–70	--

Once collected with the Teflon cylinder, the air samples were injected directly into the GC-IMS device and, from each measurement, a three-dimensional spectrum (Figure 2) was produced. All the obtained spectra were analysed, compared, and processed with LAV software (version 2.2.1. - G. A. S. Dortmund). To do so, all the analytes were carefully marked and the values of intensity, drift time and retention time of each compound were exported for posterior processing, namely, for identification purposes.

### VOCs assessment

In order to assess completely the information provided by the GC-IMS about the VOCs, all three coordinates of the spectrum were processed. In specific, the drift and retention times were processed for identification purposes and the intensity for quantification purposes.

As mentioned before, two different analytes cannot have the same drift and retention time values, in this way, the VOCs detected in the air samples were identified by comparing both these times with the values registered in a pre-developed database.

**Figure 3.**
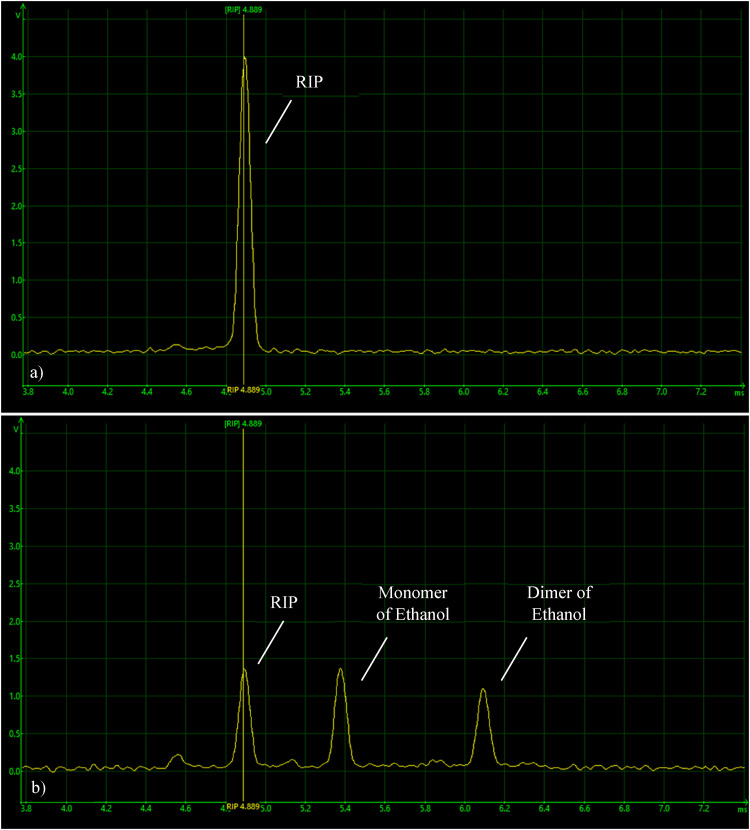
a) IMS spectra for a VOCs-free sample of air. b) IMS spectra for the detection and identification of ethanol in gaseous samples, with the RIP and the monomer and dimer of ethanol identifiable.

To develop the mentioned library of analytes, pure samples (20 μl) of VOCs were prepared in 20 ml glass vials. Once reached the thermodynamic balance between the liquid and the gaseous parts in the interior of the vial, at room temperature, 2** **ml of headspace were collected with a syringe and a needle and inserted into the spectrometer. Once analysed, their specific drift and retention times were registered in the database. The described procedure allowed the authors to have a reliable and accurate source of information regarding a vast range of VOCs without the necessity of employing a second analytical technique for verification purposes. The resolving power of the GC-IMS, ranging from 65 to 70, proved to be adequate to enable the production of evident spectra with distinctive and identifiable peaks, in this way, the identification of the pure samples and consequent development of the library of analytes were successfully achieved. Figure 3 a) and b) represent an example of IMS spectra for the case of ethanol identification. In a), the IMS spectrum in VOCs-free conditions is represented. Here, solely the RIP is visible, at 4.889 ms. In b), the IMS spectrum during the detection of both monomer and dimer of ethanol is represented. As seen, the RIP is visible at 4.889 ms and the presence of both peaks is evident and undoubtedly identifiable.

The drift and retention times of the analytes detected in the samples of indoor air were, then, crosschecked with the values registered during the database development, leading to their rapid and precise identification. To counteract eventual instrumental variations, normalized relative drift and retention times were considered.

Regarding the intensity, the third coordinate of the three-dimensional spectrum was used to plot a profile of relative intensity level for the VOCs existent in each one of the analysed locations. Such profiles enabled a direct comparison between the air composition of distinct sites and to have a perception of the concentration levels of each analyte in the original sample. Unfortunately, it was not possible to develop a calibration protocol in order to quantify the concentration of each VOC, however, the profiles of relative intensity are completely capable of demonstrating the eventual hazardousness of the detected analytes.

Initially, the average value of the intensities was calculated for all the detected peaks, and for each location. Then, the relative intensity was determined by the ratio between each average intensity and the respective intensity value in the outdoor samples, i.e., the intensity values of each VOC in the indoor air samples were normalized in relation to the intensity levels of each VOC in the outdoor air. This is equivalent to stating that the intensity of each compound in the outdoor air samples is taken as the zero value of the graphs plotted for the indoor locations. As a result, if a compound presents some intensity value above zero, that means that its intensity is higher in the studied location than it is in outdoor samples. On the other hand, if a compound intensity value is under the zero axis, this means that its intensity is lower in the location than it is in the air samples from the outside environment. To simplify the representation of the results, the relative intensities of the monomer, dimer and trimer of a specific analyte were added, and the profiles were represented in the form of a radar chart. Further details about the used procedures are given in the third chapter.

## Results and discussion

**Figure 4.**
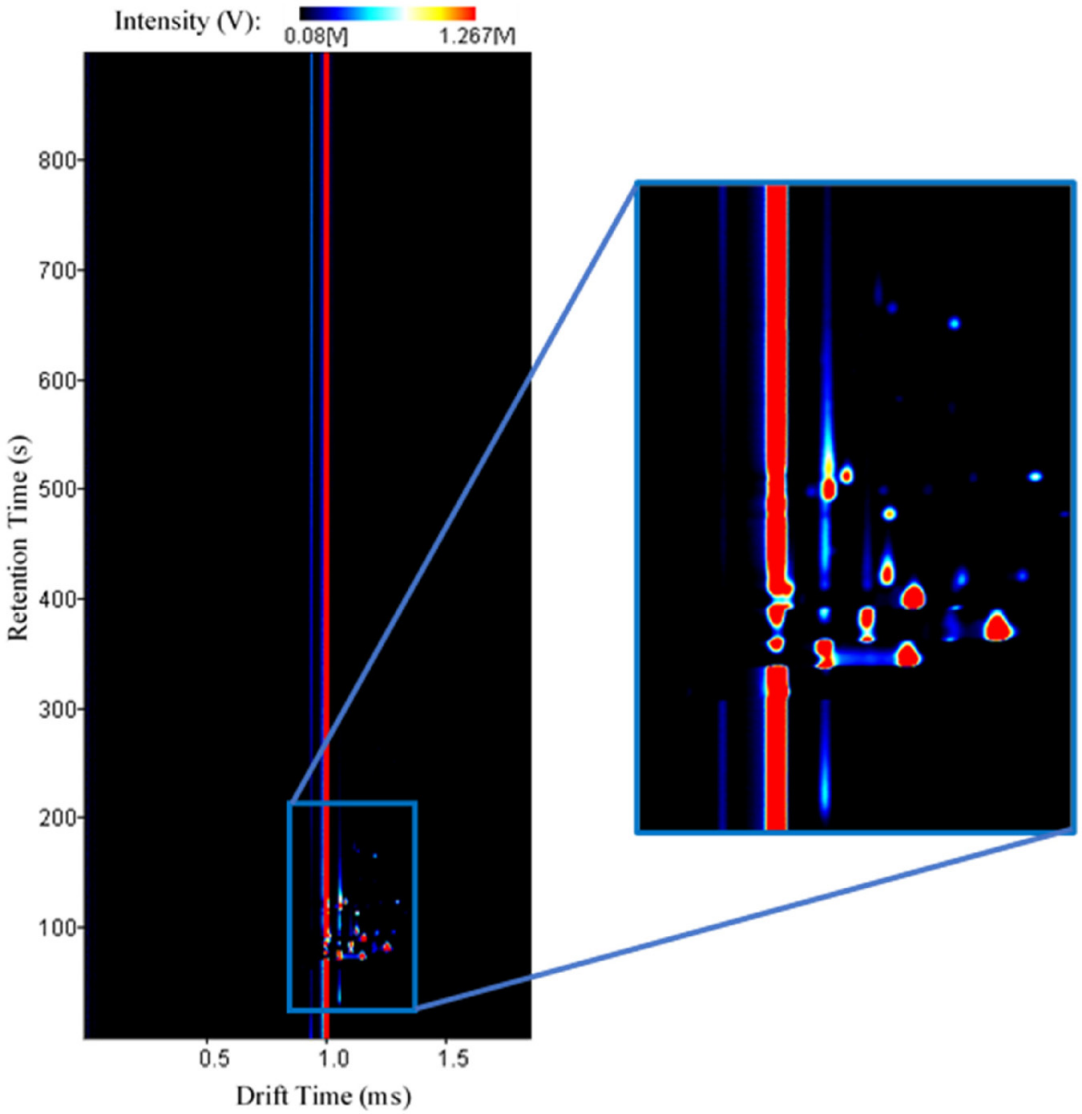
Typical GC-IMS spectrum with an enlarged section for better visualization of the detected peaks.

A total of 496 spectra were obtained from all 16 different locations (including outdoor air). In order to guarantee statistically relevant results, 31 samples were collected, during 2 consecutive days, from each location. One of the obtained GC-IMS spectra is represented in Figure 4. Here, the drift (ms) and retention (s) times are represented on the x- and y-axis, respectively. The compounds**’** intensity (V) is illustrated by a colour grade ranging from less intense (dark blue) to more intense (red). An enlarged section is also included for better visualization.

**Figure 5.**
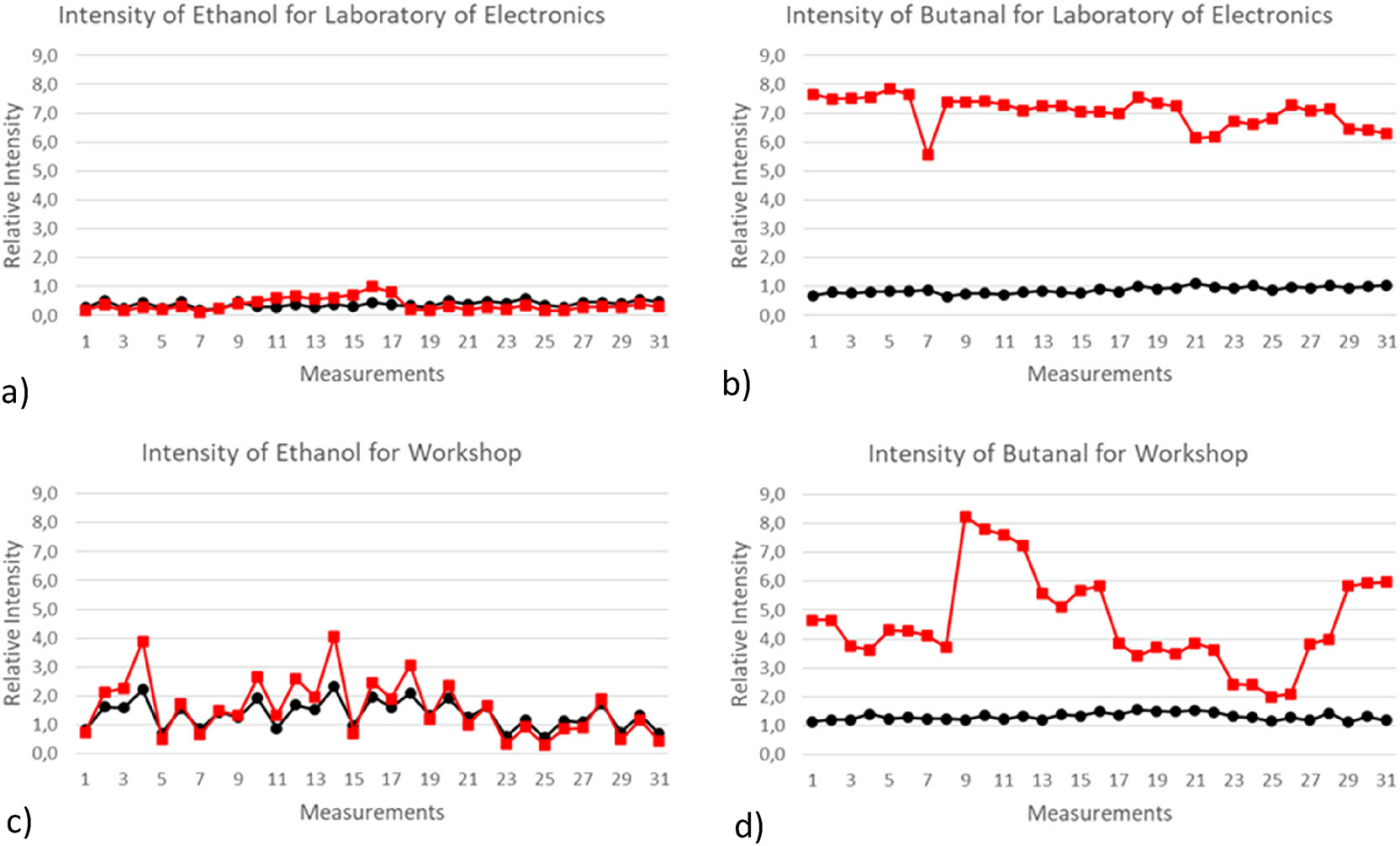
Relative intensity for the 31 measurements of the same location: a) relative intensity of ethanol for the laboratory of electronics, b) relative intensity of butanal for the laboratory of electronics, c) relative intensity of ethanol for the workshop, d) relative intensity of butanal for the workshop.

In order to analyse the variability of each location and the stability of the measurements, the intensity variation of each analyte along the 31 measurements was plotted and analysed, as shown in Figure 5: a) - d). For logistic reasons, only the graphs of two compounds (ethanol and butanal) for two locations (laboratory of electronics and workshop) were included in this work (the remaining are available upon request). The monomer and dimer are respectively represented by black lines with circles and red lines with squares.

From the plots, it can be observed the good stability of both ethanol and butanal throughout all the 31 measurements, for the same location (Figure 5 a) and b)). For the second location, however, there is some significant variation in the intensity levels of the analytes (Figure 5 c) and d)). The remaining locations and analytes presented an intermediary variation between these two scenarios, in this way, the laboratory of electronics and the workshop are, respectively, the most and less stable sites, regarding the indoor air composition. These results enabled the authors to conclude that most of the analysed locations and most of the detected VOCs are considerably stable regarding the composition of the indoor air so, the acquired data was statistically significant in order to proceed with its analysis.

Since the collected samples proved to be statistically coherent, the next step of the study consisted in identifying the analytes. As mentioned, all the spectra were analysed with a data processing software (LAV software) and, among monomers, dimers and trimers, a total of 31 peaks were found for all the locations. From these 31 detected analytes, it was possible to identify 23 peaks that correspond to 13 distinct VOCs. The identified VOCs were ethanol, 2-propanol, propanal, acetone, propanol, butanal, acetic acid, ethyl acetate, 2-butanone, butanol, pentanal, propanoic acid, and 2-hexanone. The identification was achieved, as addressed, by cross-checking the drift and retention times of each analyte with the developed database.

Table 2 includes drift and retention times for all 31 detected VOCs, as well as the name and CAS number for the identified ones. Unidentified compounds are marked with N. I. (an acronym for not identified). The table is sorted by retention time.

**Table 2.** Volatile organic compounds detected during the study.

#	Retention Time (s)	Drift Time (ms)	VOC	Note	CAS Number
1	73	1.056	Ethanol	Monomer	64-17-5
		1.150		Dimer	
2	82	1.104	2-Propanol	Monomer	67-63-0
		1.203		Dimer	
		1.254		Trimer	
3	84	1.058	Propanal	Monomer	123-38-6
4	90	1.012	Acetone	Monomer	67-64-1
		1.159		Dimer	
		1.205		Trimer	
5	93	1.060	N.I.	—	—
		1.207		—	—
6	96	1.123	Propanol	Monomer	71-23-8
		1.286		Dimer	
7	113	1.131	Butanal	Monomer	123-72-8
		1.333		Dimer	
8	118	1.061	Acetic Acid	Monomer	64-19-7
		1.177		Dimer	
9	119	1.049	N. I.	­—	—
10	119	1.183	N.I.	—	—
11	120	1.121	Ethyl Acetate	Monomer	141-78-6
12	123	1.227	N. I.	—	—
13	124	1.081	2-Butanone	Monomer	78-93-3
		1.299		Dimer	
14	125	1.061	N. I.	—	—
15	141	1.202	Butanol	Monomer	71-36-3
16	145	1.141	N. I.	—	—
17	166	1.206	Pentanal	Monomer	110-62-3
18	170	1.118	Propanoic Acid	Monomer	123-38-6
		1.130		Dimer	
19	242	1.197	N. I.	—	—
20	262	1.219	2-Hexanone	Monomer	107-87-9

As stressed, some of the detected compounds may be harmful to human health when their concentrations are elevated or when the exposure time is prolonged. Irritation and pruritus, allergic reactions, pulmonary diseases and carcinogenic pathologies are among the main consequences of exposure to VOCs. According to the literature, all the identified compounds provoke eye, nose, throat, and skin irritation, as well as pulmonary problems. The compounds 2-butanone, propanol, 2-propanol, butanol and acetone, for example, may cause headaches and dizziness. These compounds and 2-pentanone, ethanol and ethyl acetate are the cause of several nervous system disturbances. Some compounds lead to reproductive and genetic problems, specifically, 2-butanone, 2-pentanone, 2-propanol, butanal and ethyl acetate. Finally, two of the identified compounds are proved to be carcinogenic. Ethanol and 2-butanone can be the cause of cancer development in human beings, in cases of continuous exposure, as already mentioned. Overall, all the detected analytes deserve proper attention and their intensities in indoor air must be assessed and controlled.

Once identified the VOCs, the next step consisted of plotting the location-specific profiles for the relative intensity values. As stated, these profiles can be extremely useful tools for directly comparing the composition of distinct locations and having a perception of the hazardousness and toxicity of the analytes.

**Figure 6.**
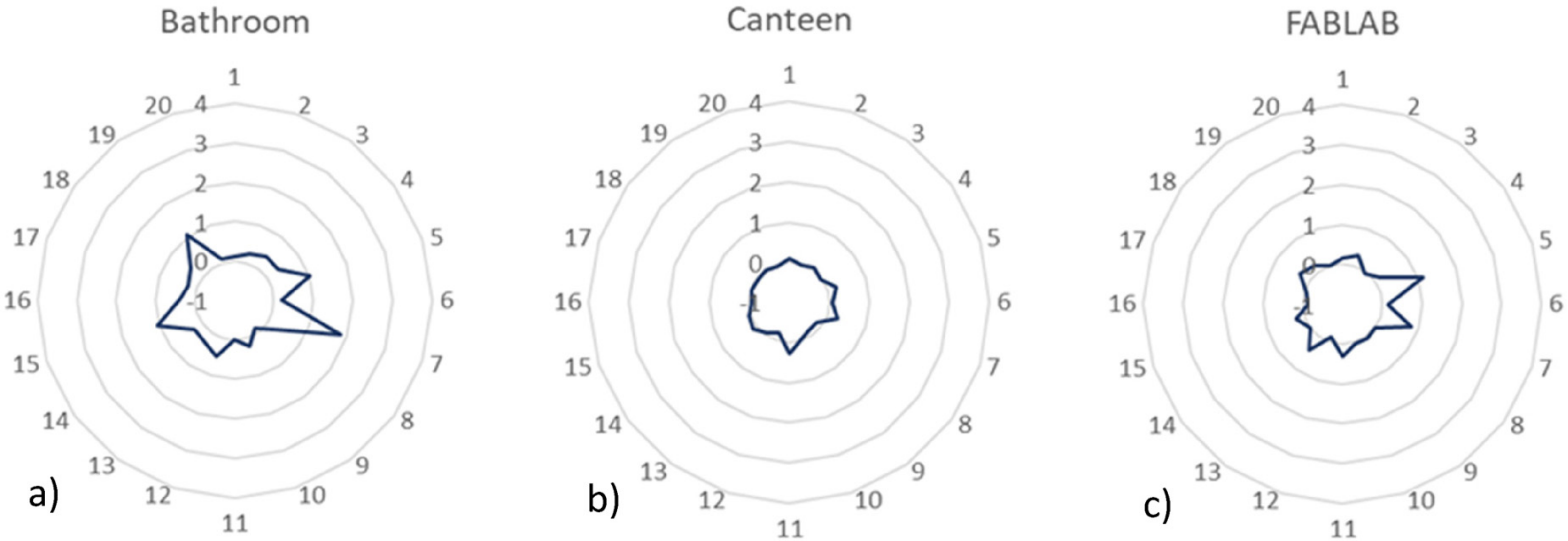
Relative intensity profiles for a) bathroom, b) canteen and c) FABLAB.

**Figure 7.**
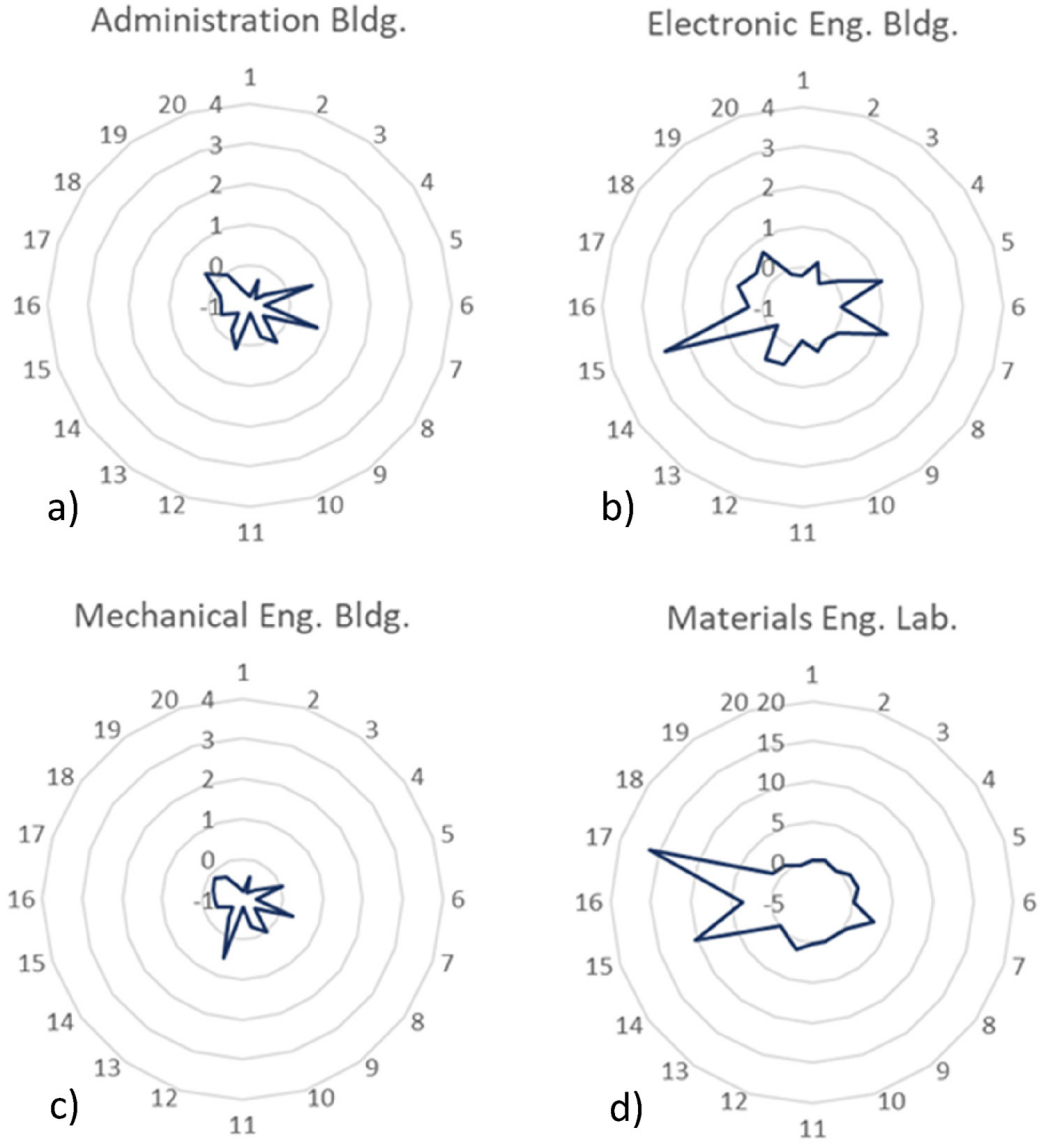
Relative intensity profiles for a) administration building, b) electronics engineering building, c) mechanical engineering building, and d) materials engineering laboratory.

**Figure 8.**
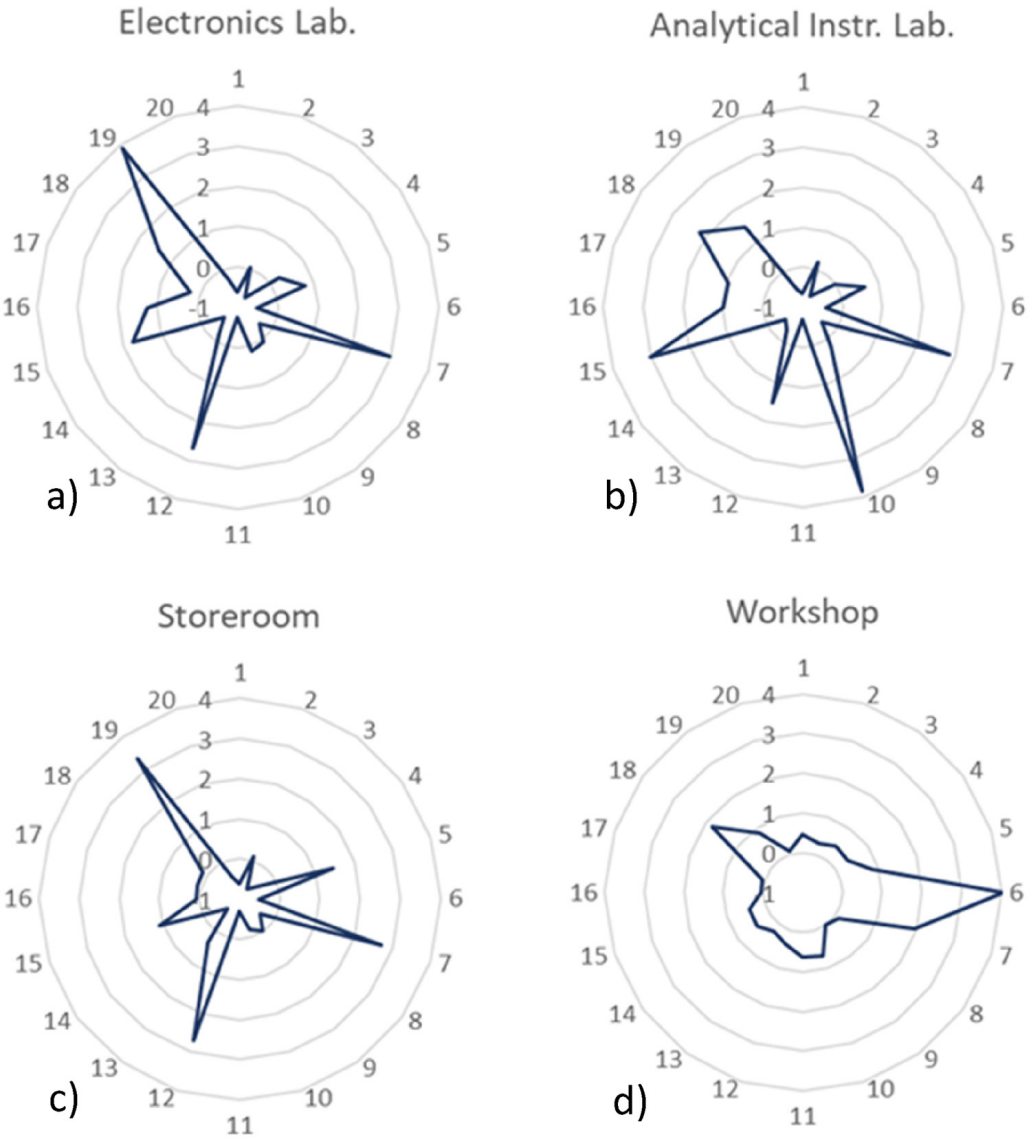
Relative intensity profiles for a) electronics laboratory, b) analytical instrumentation laboratory, c) storeroom and d) workshop.

**Figure 9.**
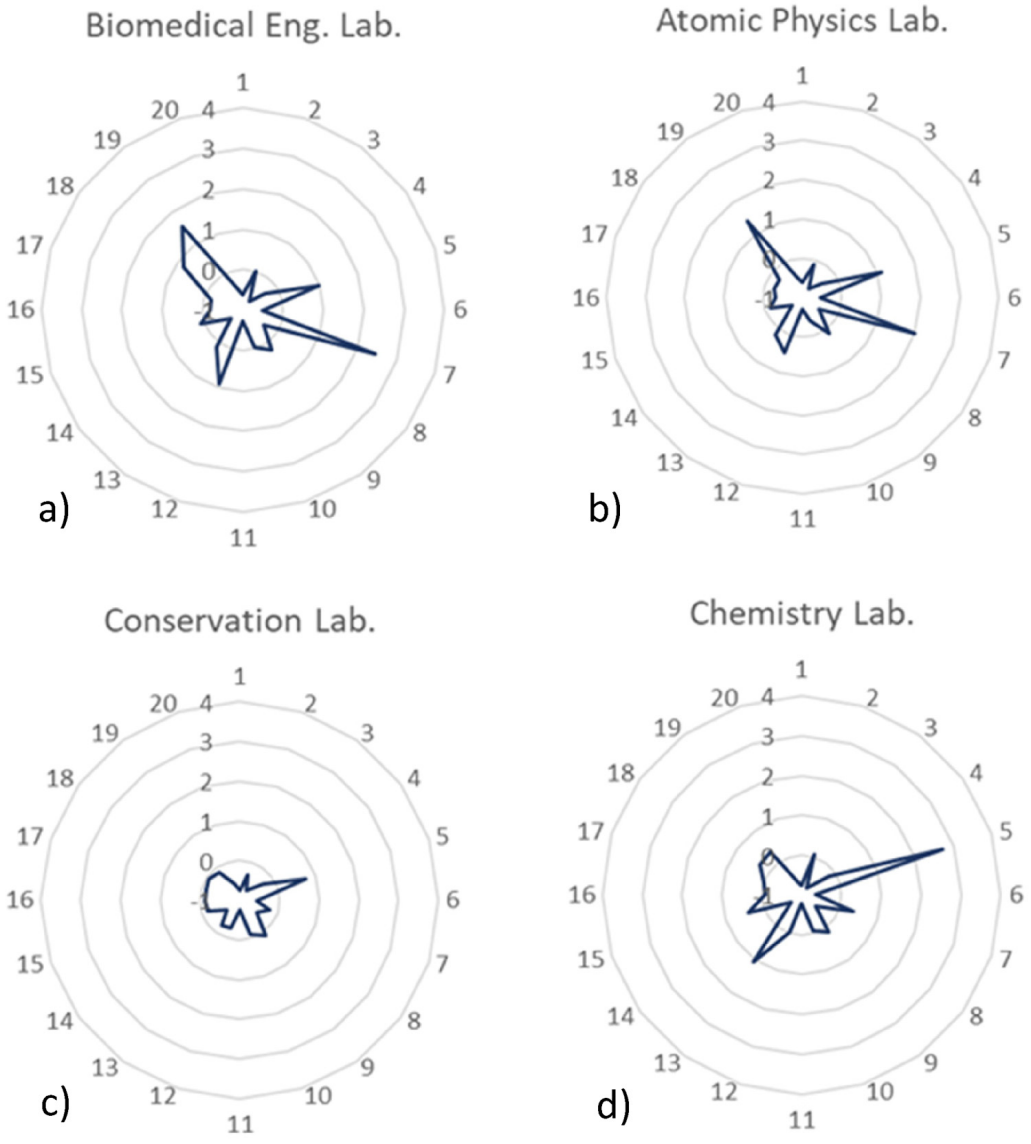
Relative intensity profiles for a) biomedical engineering laboratory, b) atomic and molecular physics laboratory, c) conservation and restoration laboratory, and d) chemistry laboratory.

The VOCs profiles of each location were represented in the form of a radar chart where the vertical axis corresponds to the relative intensity level, and the circular axis represents the compounds numeration (from 1 to 20 as represented in Table 1). To clarify, if a compound presents an intensity value of, for example, 4, it means that, with this approach, its intensity is 4 times higher in the studied location than it is in outdoor samples. Similarly, if this compound intensity is **−**4, then its intensity in the studied location is 4 times lower than its intensity in outdoor samples. Radar charts were arranged in four different groups (Figures 6 to 9).

Figure 6 illustrates the profiles plotted for the a) bathroom, b) canteen and c) FABLAB. All these three locations have profiles without considerably elevated values of relative intensity meaning that the intensities and, consequently, the concentrations of the analytes in the indoor locations are very close to the outdoor levels. Notwithstanding, some specific VOCs deserve proper attention.

Butanal (compound 7), for instance, seems to be the analyte with higher relative intensity (two times higher than in the outdoor air) in the bathroom profile and it is the second most intense in the FABLAB profile. Coincidently, compound 5 (N.I.) exhibits the highest relative intensity level in the FABLAB profile and is also one of the most evident VOCs in the bathroom profile. These facts prove that, besides the different activities developed there, there are similar sources of VOCs in both locations. some VOCs are simultaneously worth attention in the FABLAB and the bathroom facilities.

Butanol (compound 15) and 2-hexanone (compound 20) seem to have higher relative intensity, in the bathroom air, than the remaining analytes. Butanol is common alcohol usually used in cleaning products; fact that justifies its presence in the bathroom air. A large number of paints, solvents and oils have 2-hexanone in their constitution so, the building materials are the most plausible sources of this analyte in the bathroom. Both of them can cause severe cutaneous and ocular irritation, in this way, their intensity levels must be monitored.

The canteen profile reveals that the intensity values of all its compounds are considerably similar to the outdoor levels, meaning that this location seems to be properly ventilated or to possess good air renovation systems. Overall, all three profiles are very simple and do not exhibit any specific compound with considerably high levels of intensity. None of the analytes constitutes, for now, a potential risk for human health nor can be considered hazardous.

Figure 7 exhibits four profiles; a) administration building, b) electronic engineering building, c) mechanical engineering building, and d) materials engineering building. As in the previous profiles, most of the analytes have relative intensity levels around the outdoor levels, however, the number of VOCs that require proper attention is higher than in the aforementioned group.

Regarding the administration building, butanal (compound 7) and pentanal (compound 17) are the analytes with higher relative intensity. Both these analytes are known for causing severe cutaneous and ocular irritation and, besides that, butanal can be responsible for headaches, nausea and loss of consciousness. These aldehydes are commonly released into the air by activities that require any type of burning and smoke production, like cooking, smoking and fossil fuels or wood burning.

The analyte 12 (N.I.) is the most intense compound detected in the air of the mechanical engineering building. Unfortunately, it was not possible to accurately identify it, however, due to its obvious relevance to the air quality of the location, it is mandatory to use additional procedures and techniques to successfully identify the analyte and assess its eventual hazardousness. The relative intensity levels of the remaining VOCs are similar to the outdoor levels so, for now, they do not represent a risk to human health.

In the case of the electronic engineering building, a specific analyte has a more emphatic behaviour than the remaining. Butanol (compound 15) presents the highest relative intensity and is almost three times more intense in this indoor location than it is in the outdoor samples. The compounds 5 (N.I.), 7 (butanal), 13 (2-butanone) and 20 (2-hexanone) also present relative intensities higher than the outdoor levels. All these compounds are emitted by cleaning and disinfection products, smoking, industrial activities and construction materials like paints and oils, and must be carefully controlled in order to verify if their intensity levels do not increase even further.

Finally, the materials engineering laboratory profile presents the greatest concerns in this group of profiles. The results show that butanal (compound 7), pentanal (compound 17) and butanol (compound 15) are around 5, 10 and 15 times more intense in this location than they are in the outdoor environment. These extremely elevated intensities may represent a serious health risk and corroborate the necessity of developing a calibration protocol to assess the real concentration values for verifying the real hazardousness of the presence of these three analytes in the indoor air.

The third group of profiles is illustrated in Figure 8; a) electronics laboratory, b) analytical instrumentation laboratory, c) storeroom and d) workshop.

As happened in previous profiles, butanal (compound 7) has a major role in all profiles. The relative intensity of this analyte in all four locations is around three times higher than it is outdoors. Due to the known consequences caused by long periods of exposure to butanal, namely, headaches, nausea and loss of conscience, this analyte must be carefully controlled.

Both, the electronics laboratory and storeroom have very similar relative intensity profiles. Of all the compounds, the analytes 5 (N.I.), 7 (butanal), 12 (N.I.), 15 (butanol) and 19 (N.I.) stand out from all the 20 VOCs. Unfortunately, it was not possible to identify or quantify these VOCs during the data analysis, but the elevated intensities prove their special status in the air quality assessment. Compounds 12 and 19, for example, are three and four times more intense in both the electronics laboratory and storeroom than they are in the outdoor environment so, their characterization and control are essential topics.

The analytes 7 (butanal), 10 (N.I.), 12 (N.I.), 15 (butanol) and 18 (propanoic acid) present the higher relative intensities in the analytical instrumentation laboratory. Besides the already mentioned health risks of both butanal and butanol, propanoic acid is a corrosive compound that can cause severe forms of irritation in the skin, eyes, nose, throat and pulmonary tissues. Alongside propanol (compound 6) and butanal (compound 7), propanoic acid is also one of the most intense analytes in the workshop air. Coincidently, several activities that involve the use of machinery, solvents, oils and many other chemical products, are developed in both these locations; a fact that can explain the presence of similar levels of intensity for the enumerated analytes.

The fourth and final group of profiles, namely the profiles of a) biomedical engineering laboratory, b) atomic and molecular physics laboratory, c) conservation and restoration laboratory, and d) chemistry laboratory, is represented in Figure 9.

The profiles a) and b) are very similar. The analytes with a more emphatic behaviour are the VOCs 5 (N.I.), 7 (butanal), 12 (N.I.), 13 (2-butanone), and 20 (2-hexanone). The remaining analytes present intensity levels comparable to the outdoor values, meaning that they don't represent a direct risk to human health. Coincidently, these two locations belong to the same building and are very close to each other so, their profiles**’** behaviour may be related to the similarity of construction materials, furniture and activities developed in both laboratories.

In opposition to what would be expected, the relative intensities of the VOCs existent in the air of both the chemistry laboratory and conservation and restoration laboratory are very close to outdoor levels. Considering that the laboratories of these kinds are, usually, filled with all kinds of chemicals, it would be expected to find some analytes with considerably elevated levels of intensity, however, that was not the case. Except for compound 5 (N.I.), everything else presents very low intensities so, the use of proper ventilation systems and correct use of the chemicals and devices of the laboratory may be the probable cause of such low-intensity levels.

## Conclusions

The present study introduces a simple but very precise methodology for direct determination of VOCs profile in both indoor and environmental air, in a large-scale scenario, with high levels of sensibility and specificity, and capable of providing *in-situ* results in almost real-time.

A total of 31 measurements from 16 different locations were able to prove the stability of the proposed method for collection of air samples and direct analysis without the necessity of any additional chemical or previous sample preparation. Among the 31 detected analytes, it was possible to accurately identify 23 VOCs (considering monomers, dimers and trimers). Profiles of relative intensity levels were plotted for each one of the analysed locations enabling us to accurately assess the air composition of each site and identify the analytes that represent a higher risk for human health.

Summarily, it is possible to conclude that the proposed method around GC-IMS proved to be quite suitable, efficient, accurate and rapid for *in-situ* direct analysis and profiling of VOCs even at trace concentration levels. An additional study is required, especially concerning the GC-IMS database development to increase the identification of specific compounds, as well as the implementation of quantitative analysis. On another side, the proposed methodology has promising results and it can be very useful for indoor air quality monitoring and closed habitats controlling. It may seem to be negligible, however, this work represents a step forward in the field of air quality control and human health preservation.

## Notes

1. For reasons of confidentiality, the name of the chosen campus is not indicated.
